# The role of the oral microbiome in obesity and metabolic disease: potential systemic implications and effects on taste perception

**DOI:** 10.1186/s12937-023-00856-7

**Published:** 2023-05-27

**Authors:** Imke Schamarek, Lars Anders, Rima M. Chakaroun, Peter Kovacs, Kerstin Rohde-Zimmermann

**Affiliations:** 1 Helmholtz Institute for Metabolic, Obesity and Vascular Research (HI-MAG), Helmholtz Center Munich at the University Leipzig and the University Clinic Leipzig, AöR, Liebigstraße 20, 04103 Leipzig, Germany; 2 Department of Medicine III, Division of Endocrinology, Nephrology and Rheumatology, University of Leipzig, Liebigstraße 20, 04103 Leipzig, Germany; 3 The Wallenberg Laboratory, Department of Molecular and Clinical Medicine, Institute of Medicine, Sahlgrenska Academy, University of Gothenburg, 41345 Gothenburg, Sweden; 4 Deutsches Zentrum Für Diabetesforschung, 85764 Neuherberg, Germany

**Keywords:** Oral microbiome, Obesity, Taste perception, Inflammation, Metabolic disease

## Abstract

Obesity and its metabolic sequelae still comprise a challenge when it comes to understanding mechanisms, which drive these pandemic diseases. The human microbiome as a potential key player has attracted the attention of broader research for the past decade. Most of it focused on the gut microbiome while the oral microbiome has received less attention. As the second largest niche, the oral microbiome is associated with a multitude of mechanisms, which are potentially involved in the complex etiology of obesity and associated metabolic diseases. These mechanisms include local effects of oral bacteria on taste perception and subsequent food preference as well as systemic effects on adipose tissue function, the gut microbiome and systemic inflammation. This review summarizes a growing body of research, pointing towards a more prominent role of the oral microbiome in obesity and associated metabolic diseases than expected. Ultimately, our knowledge on the oral microbiome may support the development of new patient oriented therapeutic approaches inevitable to relieve the health burden of metabolic diseases and to reach long-term benefits in patients´ lives.

## Introduction

Obesity and associated metabolic disease have reached alarming levels, yet mechanisms, which drive these pandemic diseases, need further elucidation [1]. While the gut microbiome has been identified as a key player, the oral microbiome has received less attention in the context of obesity and metabolic disease, although it comprises the second largest microbiome niche of the human body after the gut [2–4]. In fact, bacteria found in the oral cavity account for 26% of all bacteria residing in the human body, whereas another 29% are located in the gastrointestinal tract [4]. Numerous studies demonstrated significant differences in oral microbiome composition between normal-weight individuals and individuals with obesity [5–14]. Most of them identified microbial signatures in the oral cavity that to a great extent paralleled obesity-associated microbiota of the gut [5, 7, 8]. Thus, some studies demonstrated an increased abundance of Firmicutes, a higher Firmicutes/Bacteroidetes-ratio and reduced microbial diversity of the oral microbiome in obese individuals [5, 7, 8, 15–17]. Longitudinal studies demonstrated, that certain oral bacteria are associated with weight gain, supporting speculations that oral bacteria may be involved in pathways leading to obesity [6, 18–21]. Studies conducted in dental medicine revealed, that the oral microbiome of individuals with obesity is characterized by an increase in traditional periodontal pathogens which reflects a well-established association between periodontitis and obesity [12, 22–25]. This association is supposed to be bidirectional; obese individuals are at greater risk to develop periodontitis and vice versa, periodontitis increases the risk for obesity and metabolic disease [6, 18, 25, 26]. Mechanisms by which oral bacteria are connected with obesity and metabolic disease might include changes in inflammatory tone, an impact on gut microbiome composition as well as other metabolically active organs, with adipose tissue in particular [6, 27–30]. Beyond that, an obesity-associated signature of oral bacteria may contribute to changes in taste perception commonly observed in obesity and metabolic disease [31, 32]. Lingual taste cells process the earliest signal in the perception of taste and have been identified as an obesity target organ [31]. Thus, adipokines, hormones derived from adipose tissue, directly regulate taste perception via corresponding receptors expressed in lingual taste cells and reduced taste bud abundancy in obese individuals appears to be a consequence of chronic low-grade inflammation characteristically found in obesity [31, 33]. Oral microbiome composition may comprise a novel factor above and beyond effects of metabolic, hormonal and inflammatory dysregulation in obesity, that impacts taste cell signaling and renewal, which in turn contribute to reduced taste perception usually observed in obesity, possibly driving food consumption, caloric intake and ultimately, weight accumulation [6, 9, 29]. This review aims to summarize a growing body of research connecting the oral microbiome with mechanisms relevant in the development and maintenance of obesity and associated metabolic disease.

### The oral microbiome

**Fig. 1:**
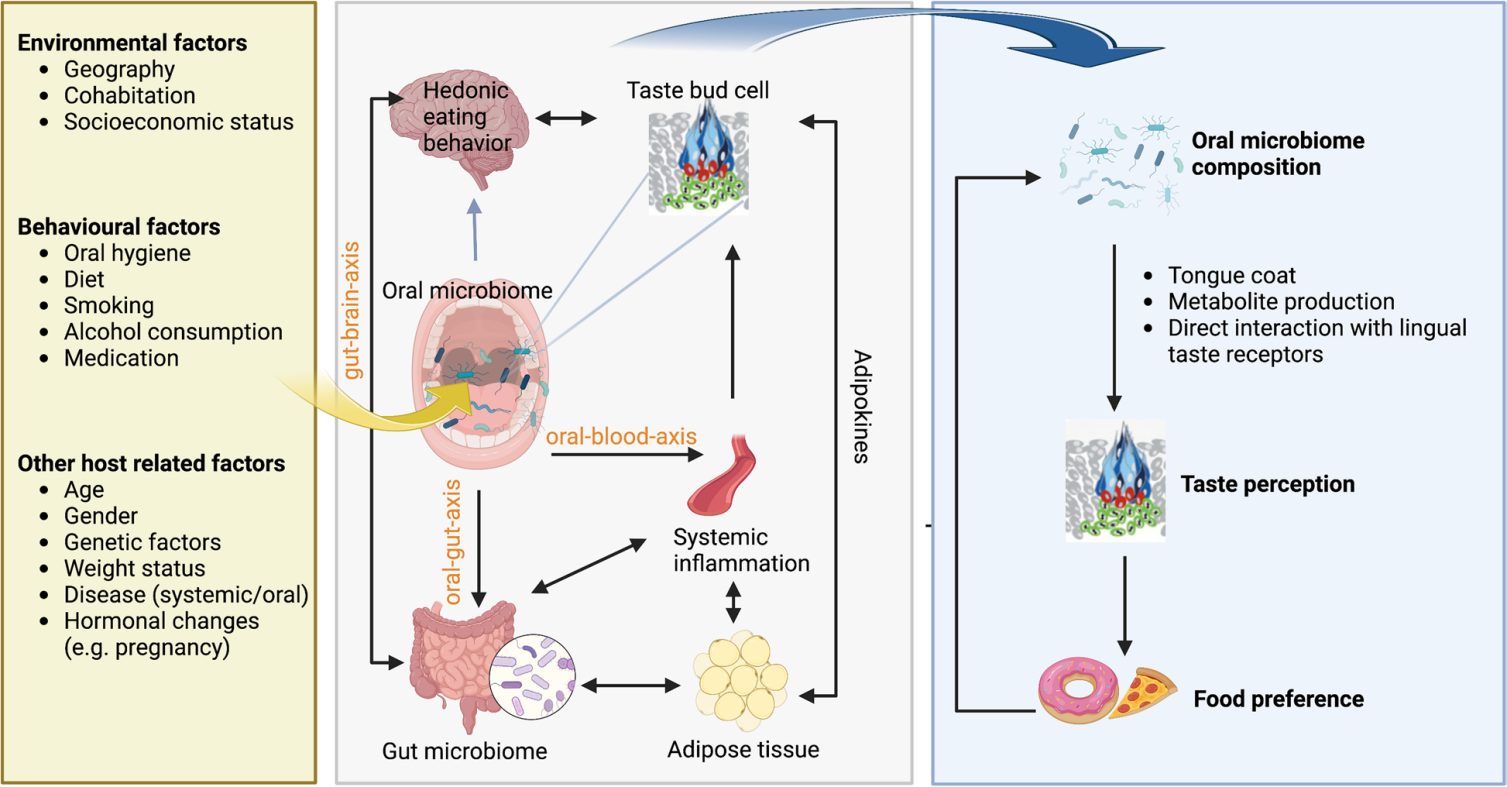
The potential role of the oral microbiome in obesity and metabolic disease and its effects on taste perception. The oral microbiome may contribute to the development of obesity and metabolic disease in various ways (grey box). Oral bacteria translocate to the gut (oral-gut-axis) potentially impacting well established effects of the gut microbiome on metabolic and inflammatory dysregulation in adipose tissue, systemic inflammation and eating behavior (gut-brain-axis) in obesity. Distant effects of oral bacteria on adipose tissue may also result from translocation of oral bacteria via the bloodstream (oral-blood-axis). Systemic inflammation, to which oral bacteria may contribute via the oral-blood-axis and through effects on the gut microbiome and adipose tissue, impacts taste bud renewal and in turn taste bud density, with potential consequences for taste perception. Taste perception is also affected by adipokines derived from adipose tissue. While the gut-brain-axis is well established, comparable direct effects of oral bacteria on brain function with consequences for eating behavior remain to be elucidated (blue arrow) but are largely supported by the presence of oral bacteria in the brain of patients with e.g. Alzheimer´s disease. Oral bacteria directly affect taste perception at the level of lingual taste bud cells with peripheral and central effects on food preference. Peripheral effects result from tongue coat formation, metabolite production and likely direct interactions with taste receptors, as has been demonstrated in extra-oral sites. Food choice in turn impacts the composition of the oral microbiome resulting in a vicious circus with potential consequences for weight regulation and obesity (blue box). The yellow box summarizes factors that have been identified to impact oral microbiome composition in general. Created with BioRender.com

The oral microbiome is defined as the collective genome of microorganisms that reside in the oral cavity [3]. The oral microbiome is the second largest and diverse microbial community in the human body after the gut [3]. It consists of several distinctive niches including the gingival sulcus, the tongue, the cheek, the hard and soft palates, the floor of the mouth, the throat, saliva and teeth [3, 34, 35]. Each of these niches provides a unique environment created by varying degrees of nutrient and oxygen availability, mechanical stress, and salivary flow [34]. These factors impact colonization and result in distinct microbial communities [3, 34, 36]. Niches with low microbial diversity are the buccal and palatal mucosae whereas the tongue displays a more diverse microflora with a complex spatially structure also including anaerobes [3, 37]. Saliva constantly bathes all oral sites, and comprises a mixture of bacteria picked up from all niches [34]. Nevertheless, bacterial composition in saliva most strongly resembles that on the tongue dorsum which, due to its large surface area, comprises a major reservoir of bacteria [34, 38]. Approximately 700 bacteria species have been identified, which belong to 185 genera and 12 phyla, of which approximately 54% are officially named, 14% are cultivated but unnamed and 32% are known only as uncultivated phylotypes. The 12 phyla comprise Firmicutes, Fusobacteria, Proteobacteria, Actinobacteria, Bacteroidetes, Chlamydiae, Chloroflexi, Spirochaetes, candidate division SR1, Synergistetes, Saccharibacteria (TM7) and Gracilibacteria (GN02) [39–41]. The largest proportion of microorganisms comprising the communities of healthy oral cavities include: *Streptococcus, Actinomyces, Veillonella, Fusobacterium, Porphromonas, Prevotella, Treponema, Neisseria, Haemophilus, Eubacteria, Lactobacterium, Capnocytophaga, Eikenella, Leptotrichia, Peptostreptococcus, Staphylococcus*, and *Propionibacterium* [42, 43]. A subset of these bacteria is hypothesized to comprise a global “core oral microbiome” which refers to genera shared by most healthy individuals [44]. Genera most frequently associated with the core oral microbiome are *Streptococcus, Veillonella, Neisseria* and *Actinomyces* [45]. While the oral microbiome remains relatively stable between individuals and across multiple geographic locations at the genus level, it can show much greater variation at deeper taxonomic resolutions [35]. Thus, diversity in the oral microbiome is not only site specific but also shows a considerable individual variability [3]. Due to their exposed location, bacteria in the oral cavity are subject to countless behavioral and environmental factors that shift oral microbiome composition and shape an individual´s oral microbiome. These factors include personal hygiene, diet, smoking, alcohol consumption, geography, cohabitation and socioeconomic status [45–47]. Beyond those, host genetics, obesity, age, pregnancy and variability in levels of host defense mechanisms are associated with a shift in oral microbiome composition (Fig. 1). This is a consequence of changes in pH, interactions among the bacteria and, on a larger time scale, gene mutations and horizontal gene transfer that extend new properties to the strain [3, 35, 45, 48, 49]. Therefore, an individual´s oral microbiome may show pronounced and potentially rapid changes in composition and activity both, spatially and temporally. Hence, the oral microbiome is dynamic in the course of its host´s development [3]. It usually helps to maintain oral health of its host, and both share a symbiotic relationship. However, any deviation from this symbiotic balance between the host and the microbiota may result in oral as well as systemic disease, including obesity and metabolic disorders [3, 46, 47].

### Oral bacteria, adipose tissue inflammation and metabolic consequences

Obesity is characterized by a chronic state of low-grade inflammation, which has been identified as an important link connecting obesity to metabolic disease [50–52]. Thus, several inflammatory pathways have been implicated in the regulation of metabolic homeostasis [52]. This interaction has been termed metabolic inflammation or “metainflammation” and has been observed in various metabolic tissues, with adipose tissue in particular [52]. Supporting the association between inflammation and metabolic dysregulation, anti-inflammatory treatment is associated with metabolic improvement and weight reduction reduces systemic inflammation in individuals with obesity [30, 53–56]. Characteristic changes of the gut microbiome underscore a causal link between inflammation and the merging obesity [57, 58]. Dysbiosis of the oral microbiome may contribute to inflammatory changes in obesity and metainflammation in adipose tissue with consequences for metabolic dysregulation [30]. Endo et al. (2010) identified adipose tissue as a target of periodontitis-associated systemic inflammation. They observed an upregulation of mRNA levels for *C-reactive protein* (*CRP*) and *interleukin 6* (*IL-6*) in adipose tissue in obese but not lean mice with periodontitis. Similar results were observed in the liver with an increased expression of *tumor necrosis factor alpha* (*TNFα*) and *CRP* mRNA. The authors concluded that periodontitis-associated systemic inflammation may exacerbate obesity associated inflammation in adipose tissue as well as in the liver through increased macrophage invasion. This in turn can contribute to metabolic dysregulation eventually resulting in metabolic disease. However, exact causal mechanisms remain uncertain, given the cross-sectional study design and as histological changes in adipose tissue were not investigated [30].

Others reported that experimental induced periodontitis resulted in an aggravated development of local white adipose tissue inflammation and systemic insulin resistance in obese rats compared to lean rats [59, 60]. Intravenous injection of sonicated *Porphyromonas gingivalis* (*P. gingivalis*) resulted in altered endocrine function of brown adipose tissue in mice. The authors concluded that endotoxemia by *P. gingivalis* potentially affects obesity by disrupting brown adipose tissue function [61]. Beyond that, mice fed a high fat diet and injected with sonicated *P. gingivalis* exhibited impaired glucose tolerance and insulin resistance along with increased liver steatosis compared to saline injections. Induction of endotoxemia also caused an increase in body weight and an accumulation of both, subcutaneous and visceral fat [56]. Intriguingly, intravenously injected sonicated *P. gingivalis* changed the gut microbiota and decreased bacterial diversity, although sonicated *P. gingivalis* never directly reached the gut. Of note, in this study there were no lipopolysaccharides (LPS) detected in the plasma of *P. gingivalis* injected mice, which by the authors was suspected to be the consequence of immediate binding to LPS-binding proteins [62]. In a different work, oral inoculation of *P. gingivalis* promoted macrophage infiltration into adipose tissue, induced elevation of serum inflammatory parameters and was associated with increased insulin resistance, although no bacteria were detected in the blood. Based on changes observed in the gut microbiome and significantly decreased gene expression of tight junction encoding proteins in the ileum, the authors interpreted systemic effects as a consequence of swallowed oral bacteria effecting the gut microbiome [63]. Although mechanistic investigations in humans are still lacking, epidemiological studies support the association between oral dysbiosis and metabolic dysregulation [64–69]. In line with this, previous studies demonstrated structural shifts in the oral microbiome of patients with diabetes compared to healthy controls [64, 66, 70]. Further, oral dysbiosis and periodontal disease associate with systemic inflammation contributing to aggravation of hyperglycaemia [66, 70]. Taken together, there is a strong evidence for oral bacteria acting as an upstream trigger of adipose tissue inflammation and consecutive metabolic disease. In the current literature two approaches are discussed which connect oral bacteria with inflammatory and metabolic effects in distant organs outside the oral cavity. First, translocation of oral bacteria into the gut (oral-gut-axis) with effects on gut microbiome composition [46, 71, 72]. Second, translocation of oral bacteria and inflammatory molecules, into the bloodstream (oral-blood-axis) [46, 72].

### The oral-gut-axis

Increasing evidence suggests, that the oral and the gut microbiome might interact to a greater extent than expected. The term “oral-gut-axis” has evolved to describe this inter-organ microbial connection [73]. The gastrointestinal tract (GI) directly connects the oral cavity with the gut in a physical and a chemical manner [73]. Transmission occurs through swallowing nutrients, drinks and saliva of which the latter is an enormous reservoir of bacteria [74, 75]. It is estimated that one gram of bacteria (10^11^) is swallowed with 500–1500 ml of saliva produced each day [6, 76, 77]. The acidic environment of the stomach and the small intestine is widely believed to act as an “oral-gut-barrier” hindering bacterial translocation along the GI tract. This assumption is supported by an over-representation of oral bacteria in the gut whenever the oral-gut-barrier is disrupted as has been observed after prolonged usage of proton pump inhibitors, gastric bypass surgery and in mouse-models with genetically increased gastric pH [73, 78–80]. However, more recent research contradicts the physiological relevance of the oral-gut-barrier [74, 81]. Schmidt et al. (2019), studied salivary and fecal microbial strain populations of 310 species in 470 individuals. They confirmed transmission of oral bacteria to the intestine with subsequent colonization to be common and extensive among healthy individuals. They further demonstrated that the vast majority of oral species are transferable although a high degree of variation between individuals existed. In more detail, approximately one in three classifiable salivary microbial cells colonized in the gut, and account for at least 2% of the classifiable microbial abundance in feces. The authors speculated that true levels of transmission are most likely even higher, as estimates in the study were rather conservative due to strict thresholds and detection limits of metagenomic sequencing. They concluded that virtually all known oral species can translocate to the intestine and that the oral cavity comprises an endogenous reservoir of bacteria potentially shaping the gut microbiome [74]. Although this study lacked culture-based analysis, others confirmed translocation of viable oral bacteria into the intestine [82–85]. The oral-gut-axis was further supported by Segata et al. (2012) reporting that oral cavity and gut bacteria overlapped in nearly half of the subjects in the Human Microbiome Project [85]. Liu et al. (2019) demonstrated that oral bacteria ectopically colonize the gut and profile the gut microbiome in germ free mice [81]. Others demonstrated that in mice oral application of *P. gingivalis* not only altered gut microbiome composition, but also effected metabolite production, which was suggested to comprise a mechanism by which oral bacteria impact metabolic disease [86]. An implication of the oral-gut-axis in metabolic disease has been demonstrated in 150 patients diagnosed with non-alcoholic fatty liver disease (NAFLD) via liver-biopsy. Significantly higher levels of *P. gingivales* were detected in their gut compared to non-NAFLD controls. Further, in patients with nonalcoholic steatohepatitis (NASH), the detection frequency of *P. gingivalis* was higher than in non-NASH individuals [87]. Treatment of periodontal disease resulted in improved oral and gut dysbiosis, systemic inflammation, Model for End-Stage Liver Disease (MELD) score and cognitive function in cirrhosis. These findings demonstrate favorable effects of decreased oral dysbiosis on gut bacteria and systemic disease [88]. Similarly, in patients with liver cirrhosis, an extensive change in the gut microbiota was found to be the consequence of an extensive translocation of oral bacteria into the gut. More than half (54%) of the enriched, taxonomically assigned bacterial species in patients were of oral origin (mostly *veillonella* and *streptococci*). The correlation of the severity of liver cirrhosis with abundance of the translocated bacteria further indicated that oral bacteria other than *P. gingivalis* could also play a role in the pathology of liver cirrhosis [89].

### The oral-blood-axis

Oral bacteria, cytokines, immune cells acting in routine surveillance in oral tissue, LPS and antigens can access blood vessels in dental pulp and periodontal tissue, especially when physiological barriers are disrupted in peridontitis [46, 72, 90, 91]. Access of oral bacteria and inflammatory molecules into the bloodstream results in bacteriaemia, systemic injury by free toxins and different immune responses through soluble antigens of oral bacteria. This ultimately triggers systemic inflammation as well as local inflammation in distant sites [46, 90, 91]. Translocation of oral bacteria to distant organs via the bloodstream with subsequent inflammatory processes is supported by mounting evidence mostly conducted in dental medicine. Thus, periodontal pathogens have been linked to inflammatory disease including rheumatoid arthritis, Alzheimer disease, atherosclerosis and cardiovascular diseases [46, 92]. Existing evidence indicates that oral bacteria actually drive rheumatoid pathology. DNA from *P. gingivalis* and *Prevotella intermidia* was detected in synovial fluid of rheumatoid arthritis patients [93]. Further, *P. gingivalis* directly promotes inflammatory synovial destruction by its unique capacity to citrullinate proteins, directly inducing anti-citrullinated peptide antibodies and triggering auto-reactive T cells [94, 95]. Similarly, DNA of different oral bacteria including *Aggregatibcter actinomycetemcomitans*, *F. nucleatum, P. gingivalis, Prevotella intermedia, Tannerella forsythia* and *Stretoccocus sanguinis* has been repeatedly detected in human atheromatous plaques, in coronary artery biopsies in patients with coronary artery disease and in endarterectomy specimens from patients who underwent surgical treatment of artherosclerosis [96–99]. It is assumed that accumulation of oral bacteria at critical sites exert direct atherogenic effects through modulation of local vascular inflammation eventually resulting in plaque formation [100]. In animal models, *P. gingivalis* passed the blood–brain-barrier from gingival ulceration and directly induced neuroinflammation contributing to cognitive dysfunction [46, 101, 102]. Oral bacteria have been also detected in amyloid plaques in the human Alzheimer diseased brain and in branches of the trigeminal nerves [91, 103]. However, bacteriaemia or systemic inflammation is not consequently detected after dental procedures, tooth brushing or flossing, all of which increase the likelihood for oral micro injuries and systemic translocation of oral bacteria [101, 104]. Therefore, the oral-blood-axis and its role in systemic inflammation is still under debate [63]. Nevertheless, recent research suggests that some oral bacteria developed the capacity to hijack immune cells which enables them to travel undetected by the immune system and facilitate their translocation to distant sites [91].

In sum, evidence suggests that oral bacteria reach distant compartments of the body. Equipped with a multitude of different virulence factors they appear to be capable to contribute to local inflammatory processes in their new destination, resulting in different tissue dysfunctions and diseases [91]. Evidence strongly supports the translocation of oral bacteria to distant organs via an oral-blood-axis [46, 72, 90]. Whether this route is implicated in effects of oral bacteria observed in adipose tissue needs further research, especially as current findings are limited to oral pathogens. The translocation of oral bacteria to the gut has been demonstrated for a wide range of bacteria residing in the oral cavity which is not limited to pathogens [74]. This route of translocation has been implicated in metabolic disease and might be of particular relevance in the context of obesity, given the prominent role of the gut microbiome in obesity. Evidence suggests that oral bacteria impact the composition of the gut microbiome [74]. Therefore, oral bacteria might be involved in a wide range of effects usually attributed solely to the gut microbiome in the context of obesity and metabolic disease, which have been described extensively before [105, 106].

### Oral bacteria and taste perception

Mechanisms by which oral bacteria affect taste perception are manifold and have been described in various populations [15, 29, 107–109]. Oral microbiome communities of the tongue dorsum and saliva appear to be of greatest relevance in modulating taste perception. Both show the closest proximity to papillae lining the tongue dorsum. Beyond that, saliva interacts with molecules from food (tastants) while transporting them to taste bud cells (TBC) [110]. When tastants bind to receptors located on chemosensory TBC, different taste qualities are perceived [111]. Functional TBC categorize into three different types of cells, where Typ I cells act mainly as supporting cells [112]. Type II cells allow humans the perception of sweet, bitter and umami tastes via G-protein coupled receptors [113]. Type III cells detect sour and certain salty stimuli mainly through activation of various ion channels [114, 115]. The existence of “fatty” as a sixth taste quality is still under debate [116]. Information from TBC is transmitted via the central nervous system to the primary gustatory cortex, which seems to be located in the insula [117, 118]. Table 1 summarizes findings regarding the association between oral bacteria and taste perception. In the following section, effects of bacteria on taste sensation are summarized.

**Table 1:** Implication of oral bacteria in taste perception

Bacteria	Population	Effect on taste perception	Potential mechanisms	Reference
**Bacteria in saliva**				
*Lactobacilli*	Elderly, acutely hospitalized adults	Inverse association with sour taste*	Metabolites synthesized by bacteria increase sour threshold	[107]
Actinobacteria	Healthy adults	Inverse association with salty taste sensitivity* Inverse association with sensitivity for sweet, sour, bitter, umami taste^+^	Metabolite production Saliva flow and pH	[108]
Bacteroidetes	Healthy adults	Positive association with salty taste sensitivity^+^ Inverse association with sensitivity for sour, and umami taste^+^	Metabolite production Saliva flow and pH	[108]
Firmicutes	Healthy adults	Inverse association with sensitivity to sweet, sour, salty and bitter taste^+^	Metabolite production Saliva flow and pH	[108]
Proteobacteria	Healthy adults	Inverse association with sensitivity for salty taste^+^ Positive association with sensitivity for umami taste ^+^	Metabolite production Saliva flow and pH	[108]
Fusobacteria	Healthy adults	Inverse association with sensitivity for salty taste ^+^	Metabolite production Saliva flow and pH	[108]
Bacteroidetes Bacteroidia	Obese and non-obese children and adolescentes	Higher Bacteroidetes abundance associated with lower total and bitter taste sensitivity	Not specified	[15]
**Bacteria in tongue film**				
Actinobacteria	Healthy adults	Inverse association with sensitivity for sour and umami taste^+^ Positive association with sensitivity for bitter taste ^+^	Metabolite production Tongue film weight and pH	[96]
Bacteroidetes	Healthy adults	Positive association with sensitivity for bitter taste ^*^ and salty and sweet taste^+^	Metabolite production Tongue film weight and pH	[108]
Firmicutes	Healthy adults	Inverse association with bitter and umami taste^+^	Metabolite production Tongue film weight and pH	[108]
Proteobacteria	Healthy adults	inverse association with salty taste^+^ positive association with umami taste^+^	Metabolite production Tongue film weight and pH	[108]
Fusobacteria	Healthy adults	inverse association with salty and sour taste^+^	Metabolite production Tongue film weight and pH	[108]
*Actinomyces, Oribacterium, Solobacterium, Catonella, Campylobacter*	PROP^$^-tasters vs non-tasters	Overexpression associated with higher responsiveness to bitter taste^*^	Not specified	[119]
*Peptococcus, Peptostreptoccoccus, Parvimonas, Lachnoanerobaculum, Prevotella, Fusobacterium*	Healthy adults	Inversely associated with salty taste sensitivity^*^	Not specified	[29]
*Bergeyella, Peptostreptoccoccus,Lachnoanerobaculum*,	Healthy adults	Inversely associated with sour and sour sensitivity^*^	Not specified	[29]
*Rothia*	Healthy adults	Positive association with salty taste sensitivity^*^	Not specified	[29]
*Streptococcus mutans*	Visually impaired children	Decreased taste sensitivity to PROP (bitter)^*^	Not specified	[120]
*Bacteroides*	Obese adults	Increase in alpha-diversity in obese low-lipid tasters^*^ higher frequency in low lipid tasters positive association with lipid taste^*^	Metabolite production	[28]
*Lachnospiracea*	Obese adults	Negative association with lipid taste^*^	Metabolite production	[21]

^*^significant association

^+^trend

^$^6-n-Propylthiouracil

#### Tongue coat as a physical barrier

Tastants cannot be perceived if they are prevented from reaching taste receptors. The tongue film comprised of and created by oral bacteria of the tongues surface can act as a physical barrier limiting access of tastants to corresponding taste receptors [121]. Multiple studies support an association between tongue film and reduced taste sensitivity for sweet, sour, bitter and salty taste [108, 122–125]. Further, improvement of subjective taste and reduced recognition threshold after tongue film removal through brushing or scraping was observed in different populations, whereas the use of mouth rinse did not result in any improvement of taste perception [122–124]. In contrary to the majority of results, comparing the taste ability of individuals with and without tongue coating, instead of intra-individual comparison before and after removing the tongue coat, did not show any significant differences [107]. Diverging results might also be a consequence of a wide variance in the definition and assessment of tongue film [121, 126].

#### Taste modulation by metabolite production

Effects of bacterial metabolites on taste have long been used in food design. The implication of metabolites of bacteria hosted by consumers in taste perception, however, is a relatively new field of research in food science. Some of the metabolic pathways of bacteria used in food design are similar to those found in some oral microbes. These can potentially result in comparable effects on taste perception ultimately driving food consumption in a similar manner. Metabolites can modulate taste in different ways. First, they influence the threshold of perception of specific molecules through changes in the basal-level production of flavor-active compounds. Second, metabolites from metabolization of food compounds can directly activate taste receptors. Third, the quantity of flavor compounds can be decreased by metabolization of taste molecules into new molecules, which no longer display chemosensory properties and cannot interact with taste receptors [127]. Gardener and colleagues demonstrated that oral bacteria in vitro catabolize salivary proteins and generate metabolic profiles similar to those seen in vivo [109]. Tongue biofilm generated higher concentration of metabolites than saliva bacteria, reaching concentrations high enough in proximity to taste receptors to affect taste perception [109]. In vivo studies revealed differences between high- and low-sensitivity perceivers of an exogenous sugar stimulus in oral catabolism of this sugar stimulus with the former showing a more efficient conversion of pyruvate to lactate and the latter showing a tendency towards continual citric acid cycle activity [109]. Others reported a relationship between taste sensitivity to oleic acid and specific signatures of the salivary proteome as well as metabolome allowing to discriminate between high and low sensitivity tasters [77]. Further, an association between reduced sour perception and higher salivary counts of *Streptococci* and *Lactobacilli* was speculated to be a consequence of organic acids production by these bacteria that increase the taste threshold [107]. Others confirmed that *Streptococcus* (Firmicutes), *Lactobacillus* (Firmicutes) and *Actinomyces* (Actinobacteria) species degrade carbohydrates into organic acids while *Prevotella* (Bacteroidetes) and *Porphyromonas* (Bacteroidetes) species break down proteins into amino acids [128]. Feng et al. (2018) found higher salivary levels of organic acids (lactate, acetate, propionate, butyrate) to be associated with a higher sensitivity of all five taste qualities [108]. Associations between organic acids concentrations and taste sensitivity were more pronounced in saliva than tongue film [108]. Most likely this is a consequence of different microbial communities inhabiting the tongue and saliva. In this study, further biological variables in saliva and tongue film were associated with taste sensitivity. Interestingly, variables in saliva (flow, organic acids, proportion of Actinobacteria and Firmicutes) found to impact taste sensitivity differed from variables found to impact taste in tongue film (sugars and proportions of Bacteroidetes) [108]. Others reported specific oral bacterial signatures possibly leading to differential molecular pathways in lipid non-tasters versus lipid-tasters irrespective of nutritional status or type 2 diabetes. Nevertheless, these differences between lipid-tasters and non-tasters became also evident in obese subjects and non-lipid tasters were found more frequently in obese than normal weight subjects [129, 130].

#### Associations of bacteria and extra-oral taste receptors

Taste receptors have been found to be expressed in numerous extra-oral sites. Although their exact function at these sites is still a topic of intensive research, some associations with bacteria have been identified which might also be of relevance in the oral cavity and with regard to taste perception [131, 132]. An increasing body of research suggests that intestinal bacteria modulate taste receptor expression and some preliminary evidence links these associations to food preference as well as consumption and possibly, taste perception [131–133]. Thus, studies in germ free (GF) mice revealed an increase in intestinal taste receptor 1 member 2 and member 3 sweet taste receptors (T1R2/3) accompanied by a higher preference for sweets and a resulting increased total energy intake from sucrose. In this study, the absence of intestinal bacteria did not change lingual T1R2/3 receptor expression. Results appear to reflect a compensatory mechanism resulting in a greater sugar consumption in the absence of intestinal bacteria, which usually contribute to a more efficient energy utilisation [132]. Others reported an increased intestinal and lingual fatty acid receptor expression in GF mice, which further associated with an increased preference for lipid emulsion, while no changes were observed in lingual sweet taste receptor expression [131]. Similarly, it was hypothesized that these results mirror a two-fold compensatory mechanism following a lack of optimal metabolization of nutrients from consumed foods in the absence of gut microbiota: First, lingual cluster of differentiation 36 (CD36) expression is increased which, in contrast to other observations, in this study associated with greater fat consumption. Second, intestinal fatty acid G protein-coupled receptors (GPRs40, 41, 43 and 120) were decreased accompanied by decreased hormonal satiety signals, which again increased fat consumption. A direct association between the absence of intestinal bacteria and reduced intestinal sodium-glucose transport protein 1 receptor expression was confirmed by colonization of GF mouse intestinal tract with conventional bacteria, which reversed changes observed under GF conditions [134]. Although these studies indicate that the absence of intestinal bacteria can lead to an altered intestinal and lingual receptor expression, potential underlying mechanisms and subsequent gustatory changes remain uncertain. Immunological studies further identified extra-oral and oral taste receptor cells as a direct target of bacteria as part of host defense mechanisms [134]. Bitter taste receptor 38 (T2R38) expressed in human sinonasal cells respond to *Pseudomonas* quorum-sensing molecules by regulating mucociliary clearance and antibacterial effects through calcium-dependent nitric oxide production. These innate host defense responses of sinonasal cells were found to be modulated by genetic variation in the T2R38 receptor gene (*TAS2R38*). Thus, cells from homozygous dominant individuals (Proline Alanine Valine; PAV/PAV) elicit a greater innate defense response than cells from homozygous recessive (Alanine, Valine, Isoleucine; AVI/AVI) or heterozygous (PAV/AVI) individuals classified as 6-n-propyithiouracil (PROP) non-taster. Results suggest a protective effect of the PAV/PAV genotype [135]. Similar innate host defense responses are triggered by the same bitter taste receptor located in gingival epithelial cells (GECs) by different oral bacteria. *TAS2R38* mRNA induction in primary GECs in response to various cariogenic bacteria was genotype dependent and highest in PAV/PAV (PROP super-taster), while lowest in AVI/AVI (PROP non-taster). PAV/PAV carriers also showed the ability to induce a high level of antimicrobial substance (human beta-defensin-2) in response to some cariogenic bacteria which results in a greater protection against caries in the PAV haplotype group [136]. As signal transduction has been shown to be similar in taste cells of different locations in the human body, it seems plausible to assume that results from these studies may also be of relevance in lingual taste cells with potential implications for taste perception [135, 137]. However, studies investigating the implication of oral bacteria in lingual taste receptor expression or their direct interaction have not been conducted. Taken together, bacteria interact with taste receptors in various ways, hence can modulate taste perception [131, 132, 136, 137]. As TBC on the tongues surface are mainly responsible for an individual´s perception of different taste qualities, oral bacteria play a key role in this interaction given their close proximity [34, 111]. A limited number of studies confirms that changes in taste perception associate with oral microbial composition and further associate with differences in habitual food consumption [9, 29]. Thus, subjects hyposensitive to salty taste, reported a more frequent consumption of bakery and salty baked products, legumes, and soft drinks than hypersensitive subjects. Subjects hyposensitive to sweet taste reported consuming more frequently sweets and desserts than the hypersensitive group. Hypersensitivety to bitter taste was associated with higher total energy and carbohydrate intake. Beyond that, some bacterial taxa on the tongue dorsum were associated with vegetable-rich (e.g. *Prevotella*) or protein/fat-rich diets (e.g. *Clostridia*) [29]. In a study investigating dental caries in children, decreased taste sensitivity to PROP was associated with higher counts of *Streptococcus mutans*. Further, dietary preferences indicated tasters were sweet dislikers, and non-tasters sweet likers [120]. In sum it can be speculated that characteristic changes in oral microbiome composition in obesity can impact taste perception and herein food intake with consequences for weight regulation.

#### Taste bud loss by inflammatory processes

The number of taste buds influences the ability to taste and was shown to be reduced in men and mice with obesity [33, 138]. Moreover, inflammatory processes were identified to be the reason for the proposed loss of taste buds [138]. Acute intraperitoneal injection of LPS reduced taste cell turnover by inhibiting cell proliferation of progenitor cell population in taste buds [138, 139]. Kaufmann et al. (2018) supported these data by finding that low-grade inflammation arising from obesity, causes elevation in TNFα level which was related to lower abundance of taste buds in mice compared to mice without obesity. By using a *Tnfα* deficient mouse, they found no changes in the amount of taste buds after high fat diet induced obesity suggesting the taste bud loss is consequence rather than cause for obesity in these mice [138]. How exactly compositional changes of the oral microbiome affect TNFα level or contribute to underlying mechanisms driving these observations is subject of intensive further research.

### Concluding remark

A characteristic signature of the oral microbiome in obesity is increasingly discussed and oral bacteria are connected to several, potentially relevant mechanisms in the development of obesity and associated metabolic diseases [5, 6, 27, 30, 31]. Oral bacteria contribute to metainflammation in adipose tissue and have been identified to target different metabolic tissues in other diseases [30, 87, 88]. Nevertheless, more research is needed to elucidate exact mechanism that connect oral bacteria with distant metabolic tissue and potential metabolic dysregulation. The oral-blood-axis as a route of translocation is promising but has been almost exclusively investigated through the lens of dental medicine, largely focusing on dental pathogens [91]. The oral-gut axis as a route of translocation is convincing and has been implicated in several metabolic diseases [74, 87, 91]. However, its implication in obesity and potential consequences of oral bacteria effecting gut microbiome composition, as a key player in obesity, need detailed elaboration. Oral bacteria also impact taste perception as a potent driver of hedonic food intake which can shape eating behavior and contribute to excessive fat accumulation [31].Vise versa, eating behavior and obesity shape the composition of the oral microbiome [45, 100]. This vicious circus underscores the central but underestimated role of the oral microbiome in the complex interaction of taste, food preference and weight regulation. The interaction of oral bacteria with oral taste cells and receptors as well as potentially associated central effects regulating food preference, possibly in a similar way as described by the “gut-brain-axis”, should receive more attention in future research. Results fuel the hypotheses, that the oral microbiome plays a more prominent role in obesity and metabolic disease than expected (Fig. 1). More research is needed to understand how these mechanisms relate to each other and to identify further implications of oral bacteria in the context of obesity development and associated metabolic disease.

## Data Availability

Not applicable.
